# Tubeless percutaneous nephrolithotomy

**DOI:** 10.4103/0970-1591.60438

**Published:** 2010

**Authors:** Madhu Sudan Agrawal, Mayank Agrawal

**Affiliations:** Department of Urology, S. N. Medical College, Agra, India

**Keywords:** Kidney stone, percutaneous nephrolithotomy, tubeless

## Abstract

**Introduction and Objective:**

Placement of a percutaneous nephrostomy tube for drainage has been an integral part of the standard percutaneous nephrolithotomy (PCNL) procedure. However, in recent years, the procedure has been modified to what has been called ‘tubeless’ PCNL, in which nephrostomy tube is replaced with internal drainage provided by a double-J stent or a ureteral catheter. The objective of this article is to review the evidence-based literature on ‘nephrostomy-free’ or ‘tubeless’ PCNL to compare the safety, effectiveness, feasibility, and advantages of tubeless PCNL over standard PCNL.

**Materials and Methods:**

We performed a MEDLINE database search to retrieve all published articles relating to ‘tubeless’ PCNL. Cross-references from retrieved articles as well as articles from urology journals not indexed in MEDLINE, were also retrieved.

**Results:**

The majority of the studies have shown ‘tubeless’ PCNL to be a safe and economical procedure, with reduced postoperative pain and morbidity and shorter hospital stay. tubeless PCNL has been found to be safe and effective even in patients with multiple stones, complex staghorn stones, concurrent ureteropelvic junction obstruction, and various degrees of hydronephrosis. The technique has been successful in obese patients, children, and in patients with recurrent stones after open surgery.

**Conclusion:**

Tubeless PCNL can be used with a favorable outcome in selected patients (stone burden <3 cm, single tract access, no significant residual stones, no significant perforation, minimal bleeding, and no requirement for a secondary procedure), with the potential advantages of decreased postoperative pain, analgesia requirement, and hospital stay. However, for extended indications, like supine PCNL, multiple, complex and staghorn stones, and concurrent PUJ obstruction, the evidence is insufficient and should come from prospective randomized trials.

## INTRODUCTION

The important milestones in the history of percutaneous renal surgery include Goodwin's description of percutaneous nephrostomy in 1955[1] and Fernstrom and Johannson's first publication of percutaneous nephrolithotomy (PCNL) in 1976.[2] Wickham in 1979 described the staged approach,[3,4] starting with percutaneous nephrostomy under local anesthesia, followed by the dilatation of the tract serially over the next few days, with subsequent stone removal under general anesthesia using a rigid 30° cystoscope. Alken used this technique as a salvage procedure to remove remaining stones after open surgery, through an operatively established nephrostomy tract.[5]

With the expanding experience of both the radiologists and the surgeons, the success rate of this procedure increased dramatically.[6,7] In 1984, Wickham described his first 100 patients undergoing one-stage PCNL,[8] where, once the puncture and dilation were complete, stone extraction was performed using an Amplatz sheath and a specially designed nephroscope. Over the past two decades, PCNL has evolved considerably, reflecting improvements in technology and surgical skill.[9–12] In recent years, ‘Mini percutaneous nephrolithotomy’ (‘mini-perc’)[13–15] and ‘Tubeless PCNL’ have been introduced with the aim to decrease the morbidity of this already established procedure.

## MINI PERCUTANEOUS NEPHROLITHOTOMY

Chan *et al*. described ‘mini-PCNL’ with a 13F nephroscope followed by the placement of an 8F nephrostomy tube with a 7F double pigtail ureteric stent.[15] Maheshwari *et al*.[16] reported lower analgesic requirement with a 9F pigtail nephrostomy tube as compared to a 28F nephrostomy tube. The smaller tube also provided a significantly shorter duration of nephrostomy tract leakage after tube removal. Several other studies have supported the use of small- bore nephrostomy tube in terms of reducing morbidity after PCNL.[17–19] However, ‘mini-perc’ suffer from the disadvantage of poorer visualization due to smaller optics and difficulty with the use of relatively delicate nephroscopic graspers.

## TUBELESS PERCUTANEOUS NEPHROLITHOTOMY

Previously it was thought that nephrostomy tubes provide hemostasis along the tract, avoid urinary extravasation, and maintain adequate drainage of the kidney.[20] However, based on the concept that the purpose of the tube is only to maintain adequate drainage of the kidney, a ‘tubeless’ approach has been developed by placing a ureteral stent or catheter to provide drainage after PCNL in lieu of a nephrostomy tube.

It may be interesting to note that the idea of ‘tubeless’ existed even in the early years of evolution of PCNL. In 1984, Wickham[8] published the results of 100 patients in which no internal or external drainage tubes were used at the conclusion of case. Authors stated that with this approach, patients could leave the hospital within 24 h and the procedure was safe and efficient with a shorter hospital stay. However, subsequently Winfield *et al*.[20] reported two patients with complications of premature nephrostomy-tube removal after the extraction of simple upper-tract calculi, who experienced serious hemorrhage and marked urinary extravasation necessitating transfusion, internal stenting, and prolonged hospitalization. They recommended that nephrostomy tube drainage should be provided during the first 24 to 48 h after percutaneous stone extraction, which subsequently became the standard practice for PCNL worldwide.

In 1997, Bellman and associates[21] challenged the requirement for the routine placement of a nephrostomy tube after percutaneous renal surgery. Their ‘tubeless’ procedure involved the placement of an internal ureteral stent without any nephrostomy tubes. The study group consisted of 50 patients, who were compared with a control group of 50 patients undergoing percutaneous renal surgery with the standard nephrostomy tube. The hospitalization time, analgesia requirements, time to return to normal activities, and cost were significantly less with this new technique.

Candela *et al*.[22] showed the cost of a ‘tubeless’ procedure to be $1,638 compared with $3,750 for traditional percutaneous surgery. Several studies in subsequent years found tubeless PCNL to be effective and safe with low morbidity that provides satisfactory results in selected cases.[23–26] In most studies, inclusion criteria for this technique were a single puncture tract, procedure lasting less than 2 h, less than three stones with a diameter <25 mm, complete extraction of all stones, and no significant bleeding at the end of the operation [Table 1].

**Table 1:** Tubeless percutaneous nephrolithotomy

Reference study	N	Mean stone burden	Postoperative drainage	Additional hemostasis used	Complications	Hospital stay (days)	Stone clearance%
Bellman *et al.* [21]	50	-	JJsN (30), JJs (20)	Nil	-	0.6	-
Delnay and Wake [23]	33	-	JJs	Nil	-	1.5	94
Limb and Bellman [24]	112	3.3 cm^2^	JJs	Nil	Pseudo-aneurysm(1)	1.2	93
Goh and Wolf [33]	10	1.8 cm	EUC(6), JJs (4)	Nil	1	2.3	80
Lojanapiwat *et al* .[ 34]	37	3.06 cm	EUC	Nil	Minor bleeding (2)	3.63	92
Karami *et al.* [36]	201	3 cm	EUC	Nil	UTI (16)	3.5	91.04
Gupta *et al*^*.*[^38]	69	1,082 mm^2^	EUC	Diathermy	Nil	1.14	97.2
Yew and Bellman [40]	4	>3 cm	Tail-stent(7F/3F)	Nil	Nil	1.5	100
Singh [65]	10	161 mm^2^	EUC	Diathermy	-	1.6	100
Yang *et al*^*.*[^67]	138	-	JJs	Nil	Nil	1.82	94.5

(N-Number of patients/renal unit; JJs-Double-J stent; JJsN-Double-J stent + Nephrostomy tube; EUC-External ureteric catheter)

## PROSPECTIVE RANDOMIZED TRIALS

To the best of our knowledge, there are only few studies in the literature comparing tubeless PCNL with standard nephrostomy drainage in randomized fashion [Table 2].

**Table 2:** Prospective randomized trials

Reference study	N	Mean stone burden	Postoperative drainage	Additional hemostasis used	Analgesia requirement	Average Hb drop (g/dl)	Stone-free rates (%)	Length of stay	Complications
Agrawal *et al*. [27]	101	3.8 cm^2^	JJs	Nil	81.7 mg MP	0.36 g%	100	21.8 h	—
Desai *et al*. [19]	10	250 mm^2^	JJs	Nil	8.5 mg D	4.2 g%	—	3.4 days	—
Feng *et al*. [28]	8	4.4 cm^3^	JJs	Nil	5.25 mg M	—	85.7	1.9 days	
Singh *et al*. [30]	30	750 mm	JJs	Diathermy	6 mg M, 415 mg D	1.2 g%	100	2.1 days	UTI (2), Stent dysuria (2)

N-Number of tubeless patients/renal unit; JJs-Double-J stent; MP- Meperadine; M-Morphine sulphate; D-Diclofenac sodium

In the largest prospective randomized trial published yet, in 202 patients treated at our center,[27] tubeless PCNL (101 patients) was found to have significant advantages over standard PCNL (101 patients) in terms of postoperative pain, morbidity, hospital stay, and period of convalescence. The average visual analogue scale (VAS) pain score on postoperative day 1 for tubeless group patients was 31 mm compared with 59 mm in standard PCNL (P <0.01). The difference in average blood loss and urinary infection for the two groups was not statistically significant. The incidence of urinary leakage from the nephrostomy site was significantly less for the tubeless group (0/101), compared with the standard PNL group (7/101). The average hospital stay in the tubeless group was less than 24 h (21.8 ± 3.9 h) and was significantly shorter than that of the standard PCNL group (54.2 ± 5 h) (P < 0.01). Tubeless group patients took 5-7 days for complete convalescence, whereas standard PCNL patients recovered in 8-10 days. No long-term sequelae were noticed in the median follow-up period of 18 months in any patient.

Desai *et al*.[19] showed definite advantages of tubeless PCNL as compared to small bore nephrostomy drainage, and conventional large bore nephrostomy drainage in a prospective randomized fashion in 30 patients. Several other randomized trials by Feng *et al*.,[28] Tefekli *et al*.,[29] and Singh *et al*.[30] have also demonstrated tubeless PCNL to be a safe and well-tolerated procedure in selected patients.

Shah *et al*.[31] compared the outcome of tubeless PCNL with small-bore nephrostomy drainage after PCNL. In this study, patients undergoing tubeless PCNL experienced significantly less postoperative pain, needed less analgesia, and were discharged 9 h earlier than patients in the other group. However, 39.4% of patients in the tubeless group had bothersome stent-related symptoms, of which 61.5% needed analgesics and/or antispasmodic agents.

In contrast, a randomized study by Marcovich and coworkers[32] showed no significant difference between a 24F re-entry tube, an 8F pigtail catheter, and a double-J stent. However, their technique involved, along with a double-J stent, the placement of a 20F Councill-tip catheter, which was removed on first postoperative day.

## PERCUTANEOUS NEPHROLITHOTOMY WITH AN EXTERNALIZED URETERAL CATHETER

Additional variations of the tubeless procedures have been described. Goh and Wolf[33], Lojanapiwat *et al*.,[34] and Mouracade *et al*.[35] reported the placement of an external ureteral stent postoperatively in tubeless PCNL [Table 1]. Compared with a control group with routine placement of nephrostomy tubes, the tubeless group with ureteral catheter had significant reduction in the length of hospitalization and postoperative analgesic requirement.

Karami *et al*.[36] reported their 5-year experience in 201 patients undergoing tubeless PCNL with only an externalized ureteral catheter, and concluded that it was a safe, effective, and economical option. Similar results were reported by Ashraf Abou-Elela *et al*.[37] in 128 patients and Gupta *et al*.[38] in a study of 69 patients [Table 1].

Gonen *et al*.[39] prospectively analyzed the outcomes of tubeless PCNLs using two different stenting techniques, externalized ureteral catheter versus double-J stent placement. They concluded that externalized ureteral catheter is as feasible as a double-J stent. Moreover, stent- related discomfort and the need for postoperative cystoscopy to remove the double-J stent can be avoided with an externalized ureteral catheter. However, they suggested that in patients who are not completely stone-free at the end of the procedure, use of a double-J stent may be more beneficial as it may help in spontaneous passage of small residual fragments.

## TUBELESS PERCUTANEOUS NEPHROLITHOTOMY WITH A TETHER

One major disadvantage of tubeless PCNL with double-J stent is the need for postoperative cystoscopy to remove the stent. Bellman *et al*.[40] suggested the placing of a 7F/3F tailed stent with an attached string exiting the urethral meatus, which can be used to pull the stent out afterward in office setting to avoid the need of cystoscopy. However this procedure has the disadvantage that some patients may remove their stents prematurely by inadvertently pulling on the tether [Table 1].

The use of tether was further modified by Bellman *et al*.[41] by placing double-J stent with its tether exiting the nephrostomy tract. This allows the stent to be removed directly from the flank in the office setting 3-12 days postoperatively by gently pulling on the tether without the need for cystoscopy.

The principle of maintaining the tether exiting from the flank may have several applications beyond routine stent placement after tubeless PCNL. In a standard PCNL, nephrostomy tube is left in when a second-look procedure is anticipated. However, it may be possible to leave only a stent with a tether in these cases as well. At the time of the second-look procedure, the tether could be used to pull the end of the stent to the level of the skin, and a guide wire could be passed antegrade into the bladder, thus reestablishing the access tract.

Berkman *et al*.[42] presented the use of the Polaris^®^ Loop stent to facilitate tubeless PCNL and minimize pain and narcotic use. The Polaris stent has two fine loops distally to minimize bladder irritation. Following PCNL, Polaris stent was placed antegradely in reverse orientation. The pigtail rested in the bladder and the loops in the nephrostomy tract with the string tether secured at the skin for simple, atraumatic removal. Authors reported that tubeless PCNL with the Polaris stent decreased postoperative pain and narcotic use, and allowed earlier discharge from the hospital.

## TOTALLY TUBELESS PERCUTANEOUS NEPHROLITHOTOMY

Totally tubeless approach was first reported by Wickham and coworkers.[8] They stated that ‘provided the kidney is stone-free, the collecting system remains intact and there is not excessive bleeding, there is no need of nephrostomy tube’. After Winfield's unsuccessful trial with totally tubeless PCNL in two cases in 1986,[20] there have been few successful reports of totally tubeless PCNL [Table 3].[43–49]

**Table 3:** Reports of totally tubeless percutaneous nephrolithotomy

Reference study	N	Length of stay (days)	Stone-free rates (%)	Transfusion rate (%)	Complications
Wickham *et al*.[8]	100	2 (mode)	94	NA	Bleeding (22%), infection (10%)
Winfield [20]	2	9	-	-	100%
Bdesha *et al*.[43]	32	2 (median)	-	Not stated	Not significant
Karami *et al*.[44]	30	1.5	90	0	Infection (2)
Aghamir *et al*.[45]	43	1.6	100	0	Not significant
Gupta *et al*.[46]	96	1.8	-	1.04	Not significant
Crook *et al*.[47]	100	2.9	76	1	One hydrothorax, 1 sepsis/bleed (horseshoes)

N-Number of patients/renal unit

In a randomized study of 60 patients, Aghamir *et al*.[50] assessed the outcome and safety of the totally tubeless PCNL in renal anomalies (horseshoe kidney, rotational anomalies of pyelocaliceal system, and ectopic kidney). The differences between tubeless and standard PCNL groups in terms of operation time, transfusion rates, complications, retreatment, and overall stone-free rate were not statistically significant. The hospitalization period, analgesia requirements, and return to normal activities were significantly less in totally tubeless group.

These studies favor the suggestion that the best available drainage of the kidney is the normal peristalting ureter. According to this school of thought, the only indication for the placement of a ureteral stent is in the situation when the ureteropelvic junction (UPJ) or upper ureter is inflamed and edematous, or where there is UPJ obstruction, managed or unmanaged. However, this approach has not formed universal acceptability due to the concerns relating to the obstruction of ureter due to clots or stone fragments. Most authors seem to favor some kind of internal drainage in tubeless procedures.

## TUBELESS PERCUTANEOUS NEPHROLITHOTOMY WITH THE PATIENT IN SUPINE POSITION

The traditional prone position used in PCNL is difficult and risky for patients with cardiopulmonary ailments and compromised respiratory functions, and in markedly obese patients. Sometimes it is impossible for the patient to lie prone because of problems with body habitus such as ankylosing spondylitis, severe lordosis or kyphosis, or hip or lower limb contractures.

Supine PCNL, in addition to saving operating room time, has several benefits. Because the tract is inclined downward and more dependent in relation to the renal pelvis, the pressure within the pelvicaliceal system is low, and stone fragments tend to fall out spontaneously. The possibility of a stone falling into the renal pelvis and the ureter is also minimized. It also permits the patient to remain in the lithotomy position for simultaneous ureteral instrumentation if necessary.[51,52]

On the other hand, PCNL in the supine position has limitations in upper caliceal puncture as the upper pole is more medial and posterior, and concealed deeply in the rib cage. Classical prone position provides a larger surface area for the choice of puncture site and a wider space for instrument manipulation.

Rana *et al*.,[53] in a study of 184 patients undergoing tubeless supine PCNL, reported stone clearance of 84%, with a mean stone size of 3.5 cm. No vascular or splanchnic injury was observed. Total 4% patients required transfusion, and 1 patient each had a perinephric collection and pleural effusion.

## TUBELESS PERCUTANEOUS NEPHROLITHOTOMY WITHOUT STRICT INCLUSION CRITERIA

For reasons of safety, most investigators have focused on using tubeless PCNL only in selected patients with uncomplicated stones. The selection criteria for tubeless PCNL include stone burden <3 cm, a single access tract, no significant residual stones, no significant perforations, minimal bleeding, and no requirement for a secondary percutaneous procedure.

However, in recent years, the tubeless technique has been applied for the treatment of multiple stones, branching and complex stones, staghorn stones, concurrent UPJ obstruction, and collecting systems with various degrees of hydronephrosis. The technique has been successful in obese patients, children, and patients with recurrent stones after open surgery.

Shah *et al*.[54] reported the results of successful tubeless PCNL in patients with expanded indications including solitary kidneys, larger stones, deranged renal function and in patients requiring multiple access tracts, supracostal puncture, or bilateral simultaneous PCNL.

Rana and Mithani[55] reported 80% stone-free rates in tubeless PCNL in 110 patients. Mean hospital stay was 16-20 h. They also concluded that the degree of obstruction, anatomic variation of renal shape and position, solitary kidney, and elevated serum creatinine are not contraindications to tubeless PCNL.

Sofer *et al*.[56] reported the applicability of tubeless PCNL without imposing preoperative restrictions in a prospective series of 126 patients. Staghorn stones, supracostal puncture, multiple accesses, anatomic anomalies, previously operated kidneys, solitary kidneys, and operative time were not considered contraindications in this study. They performed 66 tubeless and 60 regular PCNLs and reported complication rate of 9% versus 13%, respectively.

Malcolm *et al*.[57] published a retrospective review of 42 patients (47 renal units) who were treated with tubeless PCNL for complex renal stone disease (5 bilateral, 25 total/ partial staghorn, 12 renal insufficiency, and 10 infundibular stenosis or caliceal diverticulum). Mean length of hospital stay was 2.1 days. One patient required a blood transfusion and one patient developed urosepsis.

Jou *et al*.[58] performed a retrospective study to assess the outcome and safety of nephrostomy tube-free PCNL (64 procedures in 62 patients) with calculi 3 cm or greater. An 82.8% stone-free rate was reported in this study, and they concluded that with adequate hemostasis, nephrostomy tube- free PCNL can be performed in patients with complicated urolithiasis without any increase in morbidity.

Falahatkar *et al*.[59] achieved 88.09% stone-free rate in tubeless PCNL in 42 renal units with staghorn stones requiring multiple access tracts, and reported it to be a safe procedure with no significant complications.

## TUBELESS PERCUTANEOUS NEPHROLITHOTOMY IN CHILDREN

Salem *et al*.[60] assessed the effectiveness of tubeless PCNL in 20 children with a mean age of 7.5 (4-15) years. Mean operative time was 115 (45-180) min with no significant bleeding intra- or postoperatively. Tubeless PCNL had the advantages of being less painful, less troublesome, and shortening the hospital stay of the child, as compared to a group of 10 patients with similar criteria operated with PCN tube.

## TUBELESS PERCUTANEOUS NEPHROLITHOTOMY WITH PREVIOUS OPEN SURGERY

Shah *et al*.[61] reported that tubeless PCNL was feasible in a study of 25 patients with a history of ipsilateral open renal surgery, and associated with decreased analgesia requirement and hospital stay without compromising stone- free rates or increasing complications. Exclusion criteria were patients needing more than two percutaneous tracts, significant bleeding, and a significant residual stone burden that would necessitate a staged PCNL.

## BILATERAL TUBELESS PERCUTANEOUS NEPHROLITHOTOMY

Several centers have reported their experience with bilateral tubeless PCNL.[62,63] Shah and colleagues[64] found no increase in the complication rate when comparing their series of 10 bilateral tubeless PCNLs with 10 prior procedures with nephrostomy tube.

## ‘AMBULATORY’ TUBELESS PERCUTANEOUS NEPHROLITHOTOMY

Singh and co-workers[65] performed tubeless PCNL in 10 consecutive patients under spinal anesthesia (spinal low-dose anesthesia with low-dose bupivacaine plus fentanyl). No complications were noted, and all patients were discharged home the following day. The mean time to return of S1 sensation, motor block, and walking were 183,118, and 196.6 min respectively. Regional block with tubeless PCNL speeds up recovery and shortens the length of hospitalization and the analgesic requirement.

## TUBELESS PERCUTANEOUS NEPHROLITHOTOMY IN ECTOPIC KIDNEY

Tubeless PCNL also has been performed in patients with ectopic kidneys. Matlaga and associates[66] reported their successful experience with laparoscopy-assisted tubeless PCNL in six patients with pelvic kidneys.

## TUBELESS PERCUTANEOUS NEPHROLITHOTOMY IN OBESE PATIENTS

Yang *et al*.[67] reported safe and effective tubeless percutaneous renal surgery in overweight, obese, and morbidly obese patients. They analyzed the data of 45 patients who were considered normal weight (body mass index [BMI] 18.5-25), 55 overweight (BMI 25-30), 28 obese (BMI 30-40), and 5 morbidly obese (BMI 40 or greater). A stone-free rate of 94.5% was achieved. Two patients required readmission for gross hematuria and low hematocrit. One patient required selective angiographic embolization of a pseudo-aneurysm.

## TUBELESS PERCUTANEOUS NEPHROLITHOTOMY WITH SUPRACOSTAL ACCESS

Tubeless PCNL using supracostal access was done for 72 patients by Shah *et al*.[68] The outcome of these patients was compared with that of a historic cohort of similar patients with a nephrostomy tube. Two patients in the study group and three patients in the control group had postoperative hydrothorax, all of whom, except for one in the control group, were managed conservatively.

In a study by Sofikerim *et al*.,[69] 48 patients were randomized to either an 18F reentry nephrostomy tube or a 6F double-J stent. The number of supracostal accesses was significantly higher in tubeless group (P0.02). One of the seven patients with supracostal access in the tubeless PCNL group experienced pleural effusion and was treated conservatively.

## HEMOSTASIS IN TUBELESS PERCUTANEOUS NEPHROLITHOTOMY

Two hemostatic agents have been commonly used in PCNL: Gelatin matrix hemostatic sealant (GMHS) and fibrin glue [Table 4]. Gelatin matrix hemostatic sealant forms a fine suspension of particles on contact with urine in vitro.[70,71] Fibrin glue creates a thicker mucoid material on contact with urine that fails to dissolve even after 5 days. [72] Because of experimental evidence of the lithogenic properties of hemostatic agents implanted in the collecting system, an occlusion balloon is placed in the collecting system to prevent hemostatic agents from entering and causing possible obstruction.

**Table 4:** Hemostasis in tubeless percutaneous nephrolithotomy

Reference study	N	Mean stone burden	Postoperative drainage	Additional hemostasis used	Complications	Hospital stay (days)	Stone clearance (%)
Lee *et al*.[70]	7 (CS)	-	JJs	Gelatin matrix	Nil	<2.0	83
Mikhail *et al* .[75]	43 (RS)		JJs	Fibrin glue	Seroma (5), Fever (10)	1.14	-
Noller *et al* .[76]	10 (CS)		JJs	Fibrin	Nil	1.1	80
Shah *et al* .[77]	32		JJs	Fibrin glue	Nil	1.43	92.2
Jou *et al* .[81]	51 (RS)	27 mm	JJs and penrose drain	Diathermy	Fever (5), leak (1), bleeding (1)	2.2	80
Aron *et al* .[82]	20	-	JJs	Diathermy	-	1.0	100

N-Number of patients/renal unit; RS-Randomized study; CS-Controlled study; JJs-Double-J stent

Nagele and Schilling *et al*.[73,74] reported the use of gelatine- thrombin-hemostatic sealant following mini-PCNL in a tubeless setting. Mikhail *et al*.[75] were the first to use fibrin glue as a hemostyptic sealant in 20 patients during PCNL. Several other studies have used fibrin glue with good effects.[76–78]

Aghamir and colleagues [79] used oxidized cellulose (Surgicel^®^) to seal the working tract and concluded that such sealing of the nephrostomy tract after totally tubeless PCNL did not decrease bleeding or urinary extravasation. In a prospective study of 50 patients, Singh *et al*. [80] evaluated the role, safety, and efficacy of using absorbable gelatin tissue hemosealant (Spongostan^®^) in tubeless PCNL. They observed lower wound soakage/discomfort in the gelatin-assisted tubeless PCNL group as compared to controls.

In 51 patients, Jou *et al*.[81] reported cauterization of the access tract after completing the PCNL with a double-J stenting. Aron *et al*.[82] also reported diathermy coagulation of the intrarenal bleeders and tract in 20 consecutive patients of tubeless PCNL and reported that fulguration of visible intrarenal and tract bleeders is a simple, safe, and effective hemostatic adjunct. Mouracade *et al*.,[27] in their study of 37 patients, electrocoagulated the nephrostomy tract by a blunt electrocautery loop mounted on a 26F resectoscope. The mean decrease in hemoglobin was 0.95 g/dl. No blood transfusion was required.

## ADVANTAGES OF TUBELESS PERCUTANEOUS NEPHROLITHOTOMY

- Patients who undergo tubeless PCNL have significantly less pain postoperatively and require less analgesia dosage.
- Tubeless PCNL minimizes the hospital stay, allowing many patients to be discharged from hospital in less than 24 h.
- A tubeless procedure offers the advantage of passive dilation of the ureter caused by the indwelling double-J stent to facilitate passage of any unrecognized small stone fragments.
- The omission of a nephrostomy tube with the placement of an indwelling double-J stent is associated with rapid healing and minimal urine leakage.

## DISADVANTAGES OF TUBELESS PERCUTANEOUS NEPHROLITHOTOMY

- The general consensus is that the tubeless approach is feasible only in a selected population that generally excludes cases requiring two or more accesses, significant intraoperative bleeding, or situations with a likelihood of residual stone fragments.
- The other limitations to tubeless PCNL are the possibility of missed residual stone fragment (4-5 mm, invisible on initial postoperative fluoroscopy) that become apparent later, as a tubeless operation precludes a ‘second-look’ procedure.
- The need for an additional procedure, that is, cystoscopy, to remove the double-J stent.
- ‘Stent dysuria’, which can be troublesome in some patients, and may even warrant the need of early double-J removal.
- One of the criticisms of this approach is that the ureteral stent does not necessarily provide drainage of the kidney. It is well established in animal models that stents cause a degree of obstruction and raise intrapelvic pressure.[83]

## CONCLUSION

Tubeless PCNL can be used with a favorable outcome in selected patients (stone burden <3 cm, single tract access, no significant residual stones, no significant perforation, minimal bleeding, and no requirement for a secondary procedure), with the potential advantages of decreased postoperative pain, analgesia requirement, and hospital stay.

Several retrospective studies have shown that its application can be extended even in patients with multiple, complex and staghorn stones, concurrent UPJ obstruction, solitary kidney, previous ipsilateral open surgery, raised serum creatinine level, with multiple or supracostal tracts, and in patients undergoing bilateral synchronous PCNL. The technique has been successful in obese patients, children, and patients with recurrent stones after open surgery. However, for all these extended indications, the available evidence is insufficient, and needs to be substantiated by prospective randomized trials.
